# Infantile epileptic spasms syndrome: an etiologic study of 361 patients with infantile epileptic spasms syndrome

**DOI:** 10.3389/fped.2024.1522079

**Published:** 2025-01-09

**Authors:** Linghui Zhu, Yuan Xia, Hao Ding, Tong Zhang, Jun Li, Baomin Li

**Affiliations:** ^1^ Cheeloo College of Medicine, Shandong University, Jinan, Shandong, China; ^2^ Department of Pediatrics, Qilu Hospital of Shandong University, Jinan, Shandong, China; ^3^ Children's Hospital Affiliated to Shandong University, Jinan Children's Hospital, Jinan, Shandong, China

**Keywords:** etiology, pathogenic genes, hypsarrhythmia, infantile epileptic spasms syndrome (IESS), prognosis

## Abstract

**Introduction:**

Infantile Epileptic Spasms Syndrome (IESS) typically has a profound impact on the neurodevelopment of patients. The study on IESS indicates possible geographical variation in etiology and a lack of data from China. Our study intends to summarize the etiology of IESS and analyze its characteristics.

**Methods:**

A retrospective analysis was performed to gather clinical data from patients diagnosed with IESS at the Department of Neurology of Qilu Hospital of Shandong University and the Children's Hospital Affiliated to Shandong University between June 2017 to May 2024.

**Results:**

A total of 361 patients with IESS were included, comprising 115 structural cases (31.9%), 37 genetic cases (10.2%), 32 genetic-structural cases (8.9%), 9 metabolic cases (2.5%), 3 infectious cases (0.8%), and 165 cases with unknown etiology (45.7%). No immunological cause was determined. The primary cause of the condition was linked to hypoxic-ischemic encephalopathy (HIE), with structural brain abnormalities following closely. The predominant pathogenic genes identified were *TSC2, NF1, SCN8A*, and *KCNQ2*. Male gender, preterm infants, low birth weight infants, and developmental regression in patients were associated with a higher likelihood of structural etiology. Patients exhibiting developmental regression before the commencement demonstrated inferior outcomes. Patients administered adrenocorticotropic hormone (ACTH) exhibited a higher likelihood of attaining seizure control, and those who responded favorably to the medication saw improved results.

**Conclusion:**

The predominant etiology of IESS is structural, succeeded by genetic factors, with significant pathogenic genes comprising *TSC2, NF1, SCN8A*, and *KCNQ2*. The genetic classifications exhibit geographic variability. Genetic and structural etiologies are frequently linked to an unfavorable prognosis. Genetic testing can help clarify the etiology of IESS when metabolic screening and brain MRI results are negative. The advancement of genetic testing is crucial for future targeted and individualized diagnosis and therapy.

## 1 Introduction

Infantile epileptic spasms syndrome (IESS), one of the most common developmental and epileptic encephalopathies (DEEs) of infancy and children, was first documented in 1841 by William James West reported his son's condition (1). The illness, once termed WEST syndrome or infantile spasms, was renamed by the International League Against Epilepsy (ILAE) in 2022 as Infantile Epileptic Spasms Syndrome (IESS). It encompasses patients with WEST syndrome, defined by the triad of epileptic spasms, hypsarrhythmia, and developmental regression (2), along with additional epileptic spasms occurring before the age of 2 years. Patients with IESS may have other seizure types in addition to epileptic spasms, and some may develop Lennox-Gastaut syndrome (LGS) later in life. The prevalence of IESS is approximately 0.2–0.5 per 1,000 live births (3), predominantly occurring between the ages of 3 and 12 months, with a higher incidence in males (4). The underlying cause of IESS can be identified in about 60% of patients (5). Despite the rising percentage of research on IESS with a definitive etiology, the majority of patients remain of unknown etiology. A significant number of experts, including J.P. Osborne, G. Karvelas, and S.T. Demarest, maintain that the etiology remains unidentified in at least 30% of patients with IESS (5–8). These studies have been undertaken in Europe and the United States. A limited number of the study populations were Chinese, including Li-Hong Ren and Peng P. (9, 10), and among these, only a small fraction comprised large-sample studies concerning Chinese patients with IESS, particularly in analyzing the correlation between etiology and clinical features. Considering the differences in populations of different countries and geographic locations, our study of the etiology of IESS in a large sample of patients from selected areas of China remains uniquely relevant. IESS, classified as an epileptic encephalopathy, is typically linked to mild to severe intellectual disability, significantly impacting the quality of patients’ lives (11). Clarifying the etiology of IESS patients may enhance clinical care.

The 2017 ILAE divided the etiologies into six categories, including genetic, structural, metabolic, infectious, immunological, and of unknown etiology (12). In this study, we summarized and analyzed the etiology of 361 IESS patients. To better define the etiological classification, we classified the overlap of structural and genetic causes as genetics-structural causes. According to the etiological classification, we collected clinical data including age of onset, time of intervention, type of seizure, type of electroencephalogram, treatment, and prognosis, and explored the distribution of different etiological groups according to this clinical data. This study aims to enhance our capacity to classify the etiology of IESS patients in particular parts of China and to aid in therapeutic care.

## 2 Materials and methods

### 2.1 Patients

We retrospectively collected IESS patients who attended Qilu Hospital of Shandong University and the Children's Hospital Affiliated to Shandong University from June 2017 to May 2024. We included cases with (1) meeting diagnostic criteria for IESS as established by the ILAE in 2022. Hypsarrhythmia was not a necessary condition, and multifocal spikes and spasms electroclinical seizures in addition to hypsarrhythmia were also included; (2) Comprehensive clinical data.

The studies involving human participants received approval from the Institutional Ethics Committee at Qilu Hospital of Shandong University. Written informed consent was obtained from the parents. The participants’ legal guardian/next of kin provided written informed consent to participate in this study. This study was performed in accordance with the principles of the Declaration of Helsinki.

### 2.2 Clinical data

Examination tests of the patients were collected, including brain MRI results, basic metabolic tests (plasma amino acids, urine organic acids, lactate, ammonia, homocysteine, ceruloplasmin, etc.), video EEG, and genetic testing. Among them, the EEG at the onset of the seizure was categorized as typical hypsarrhythmia, atypical hypsarrhythmia, and multifocal spikes.

In genetic testing, patients were mainly screened for genetic variants through whole-exome sequencing (WES), while some also underwent chromosomal karyotyping, copy number variation (CNV) testing, and mitochondrial genetic testing. The results were categorized as the pathogenic or suspected pathogenic variants based on the patient's clinical phenotypes. Other results underwent secondary investigation of clinical traits. The single nucleotide variations (SNVs), insertions, and deletions identified by genetic testing in this study were confirmed with Sanger sequencing. SNV, insertion, and deletion variations were confirmed by Sanger sequencing, while exon deletions or duplications were validated with PCR sequencing. Exon deletions or duplications were confirmed using PCR sequencing of the family lines. The test has specific limitations, such as that the test program only targets genes known to be associated with diseases, some genes that have not yet been specified are not included in the scope of the test, and so on. So all partial results of unknown pathogenicity need to be reviewed by a specialist pediatric neurologist. The pathogenicity of the genetic variations was evaluated by applying the American College of Medical Genetics and Genomics (ACMG) genetic variant classification criteria (13).

Clinical data from patients with IESS were collected. We recorded gender, age of onset, intervention interval (duration from the identification of spasm seizures to the initiation of ACTH or alternative ASM treatment), developmental status at the onset of spasms, family history, birth history, treatments, seizure control, and other relevant factors. Prognosis: seizure control was classified based on seizure frequency into four categories: complete remission (clinically seizure-free), effective (>50% reduction of seizures), poor effect (<50% reduction of seizures), and ineffective (no improvement or worsening of seizures compared to prior status). The classification was mostly established based on Engel's grading and the seizure reduction levels outlined in Anderson's study (14). The treatments were classified as adrenocorticotropic hormone (ACTH) and non-ACTH. EEG changes (regardless of the disappearance of hypsarrhythmia) were collected after the ACTH treatment (ACTH treatment for 14–28 days). The above information was evaluated by specialized neurological clinicians within the team.

## 3 Statistical analysis

Statistical analysis was performed using Statistical Package for Social Science (IBM, SPSS Statistics Version 25). Non-normally distributed measurements were expressed as median (range), and comparisons between groups were made using the Mann-Whitney U test. Count data were expressed as the number of cases and percentage (%), and comparisons between groups were made using the *χ*2 test or Fisher's exact test. *P* < 0.05 were considered statistically significant.

## 4 Results

This study comprised 361 individuals with IESS, comprising 223 males and 138 females, with an age of onset of 5 (4,7) months, and all of whom obtained brain MRI data. The etiology was elucidated in 196 cases (54.2%). The baseline characteristics of the patients are shown in Table 1.

1. The specific etiologic classification is presented in Table 2: Regarding the etiology of the 361 patients, 115 cases (31.9%) were structural, 37 cases (10.2%) were genetic, 32 cases (8.9%) were genetic-structural, 9 cases (2.5%) were metabolic, 3 cases (0.8%) were infectious, and 165 cases (45.7%, including 58 that were not genetically tested) were classified as unknown. No immunological cause was identified in the instances.
  (1) Structural etiology: In all patients, structural factors were 147 cases (40.4%), including 32 cases of genetic-structural, resulting in 115 cases of structural etiology. Of the total, 39 cases (33.9%) were congenital structural, whereas 76 cases (66.1%) were acquired structural. Of the 39 congenital structural causes, 37 cases (94.5%) were malformations of cortical development (MCD): 8 cases of macrogyria/polymicrogyria/Lissencephaly were documented, including 1 case was macrogyria with heterotopic; 12 cases were focal cortical dysplasia (FCD), of which, 1 case is FCD with heterotopic; 7 cases were hypoplasia of the corpus callosum; 3 cases were heterotopic, and 7 cases were unspecified MCD. Two cases were non-MCD: 1 case of Septum pellucidum hypoplasia with hydrocephalus, and 1 case of Dandy-Walker deformity.

**Table 1:** Baseline characteristics.

Variable	Total patients	Percentage
Sex		
Male	223	61.8%
Female	138	38.2%
Age of onset		
<3 months	41	11.3%
3 months−6 months	169	46.8%
6 months−12 months	136	37.7%
≥12 months	15	4.2%
Intervention interval		
<3 months	328	90.9%
3 months−6 months	28	7.7%
≥6 months	5	1.4%
Family history		
Yes	30	8.3%
No	331	91.7%
Development at the onset of spasms		
Normal	80	22.2%
Abnormal	281	77.8%
Gestational age		
Term infant	315	87.3%
Premature infant	46	12.7%
Birthweight		
<1.5kg	9	2.5%
1.5kg−2.5kg	38	10.5%
2.5–4kg	289	80.1%
≥4kg	25	6.9%
Type of initial seizure		
Spasm	348	96.4%
Spasm and myoclonus	7	1.9%
Spasm and absence seizure	6	1.7%
EEG types at the beginning of the disease		
Typical hypsarrhythmia	295	81.7%
Atypical hypsarrhythmia	24	6.7%
Burst suppression	12	3.3%
Other	30	8.3%

**Table 2:** The specific etiologic classification.

Etiologic categories (*n*)	Specific causes	Total
Structural-acquired (115)	Structural-unacquired	39
	MCD	37
	Macrogyria	4
	Macrogyria and heterotopic	1
	Polymicrogyria	2
	Lissencephaly	1
	FCD	11
	FCD and heterotopic	1
	agenesis of the corpus callosum	7
	heterotopic	3
	MCDS not clearly classified	7
	Others	2
	Septum pellucidum hypoplasia with hydrocephalus	1
	Dandy-Walker deformity	1
	Structural-acquired	76
	HIE	57
	With IVH	12
	With hypoglycemia	6
	Hypoglycemic encephalopathy	9
	Brain injury secondary	4
	Malacia and hydrocephalus of unknown origin	6
Genetic-structural (32)	TSC	15
	* TSC2*	8
	* TSC1*	2
	None	5
	*NF1*	5
	Macrogyria with TUBB2B/PAFAH1B1	2
	Other MCDS with *IARS2*/*RAB3GAP1*/1p36.33p36.32 deletion (*CHD2*)	3
	agenesis of the corpus callosum with STXBP1	1
	HIE and *KCQN2*/*RELN*/*SHANK3*/*CACNA2D3*/*RAC3*/*CHD3*	6
Genetic (37)	*SCN8A*	4
	*SCN2A*	3
	*KCNQ2*	2
	*ABCD1*	2
	*NR2F1*	2
	*UBA5*	2
	*STXBP1*	1
	*KCNK4*	1
	*CACNA1C*	1
	*GABRA1*	1
	*PRRT2*	1
	*SPTAN1*	1
	*MECP2*	1
	*CUL4B*	1
	*NARS1*	1
	*RNF13*	1
	*KCNQ5*	1
	*VPS11*	1
	*WWOX*	1
	*GRIA3*	1
	*ZEB2*	1
	*DEPDC5*	1
	5p15.2 duplication	1
	18p11.32 duplication	1
	15q11.2–15q13.3 duplication	1
	1p36.33p36.32 deletion (*GABRD*)	1
	16p133 deletion (*NPRL3*)	1
	Down syndrome	1
Metabolic (9)	Disorder of glycosylation	5
	* ALG13*	3
	* ALG2*	1
	* SLC35A2*	1
	Electrolyte metabolism disorder	
	*AVPR2*	1
	Metabolic encephalopathy	
	Leigh syndrome	3
	
Infection (3)	Intrauterine infection	
	CMV	3

MCD, malformations of cortical development; FCD, focal cortical dysplasia; HIE, hypoxic ischemic encephalopathy; IVH, intra-ventricular hemorrhage; CMV, cytomegalovirus; brain injury secondary, areas of softening due to brain tissue damage and necrosis resulting from previous craniocerebral trauma or intracranial infection.

In the 76 cases of acquired structural etiology, 66 cases (86.8%) were attributed to perinatal brain injury, of which 57 cases (86.4%) were classified as hypoxic-ischemic encephalopathy (HIE) with/without intra-ventricular hemorrhage (IVH) and hypoglycemia. Twenty-three of the HIEs were attributed to brain injury in preterm newborns, and the rest were near-full-term/full-term HIEs; Nine (13.6%) cases were classified as hypoglycemia encephalopathy. Of non-perinatal brain injuries, 6 cases were unexplained brain white matter softening/hydrocephalus, and 4 cases were secondary brain injuries.

**Figure 1:**
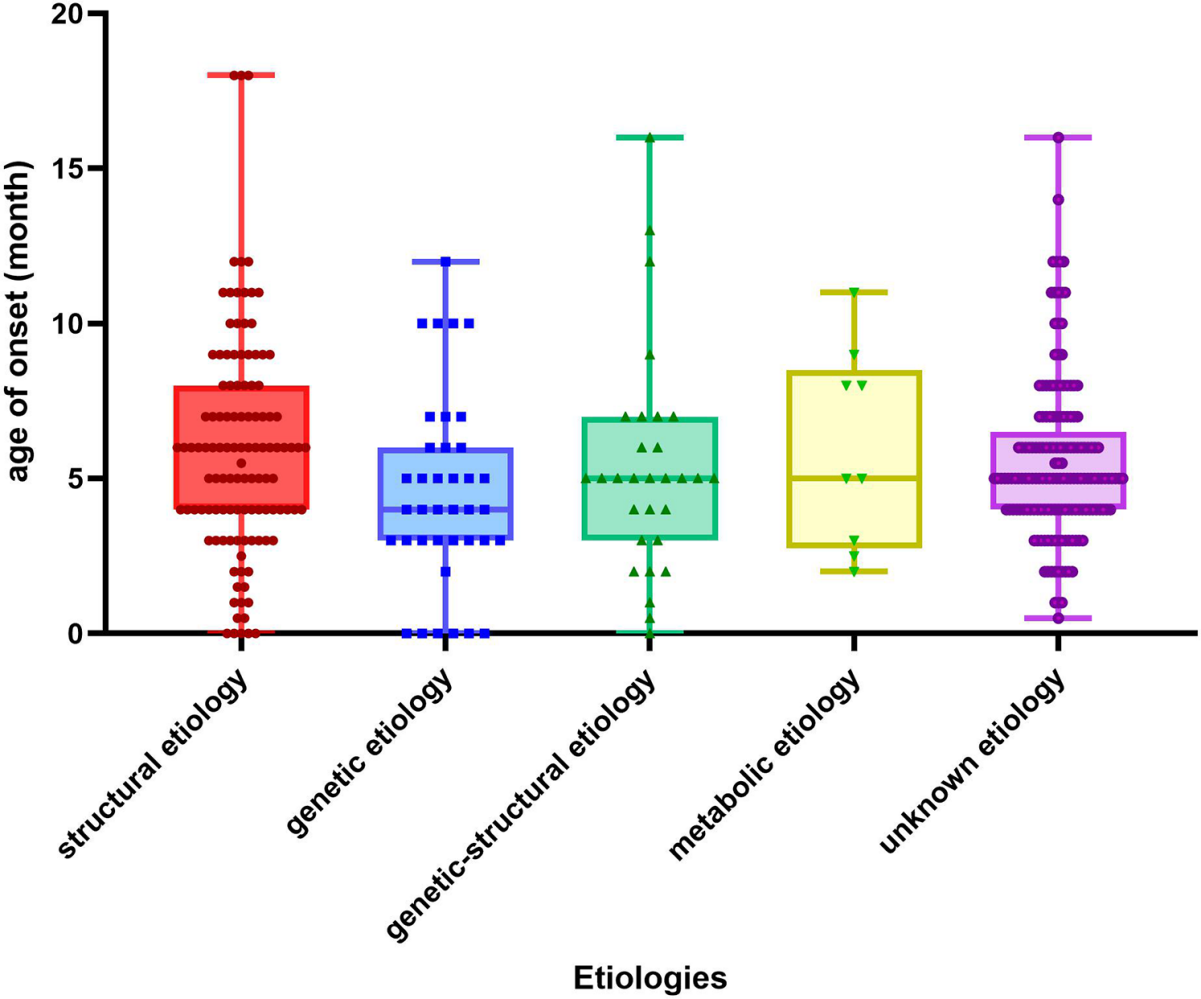
Age of onset for different etiologies. The X-axis in the chart represents the classification of etiology and the Y-axis represents the months.

(2) Genetic etiology: In the 361 cases, 210 patients (58.2%) had received genetic testing, and 210 patients completed WES testing. In that, 9 patients were also tested for CNV, 8 patients for mitochondrial genes, and 2 patients for chromosomal karyotyping. Sixty-nine patients (32.9%) of the 210 patients were determined to have a pathogenic factor, 30 patients had monogenic variants, 7 patients had chromosomal variants, 1 patient had a mitochondrial variant (IESS-associated variant could not be determined), and 32 patients also had structural factors. Among them, the most common genes in single gene variants included *SCN8A* (4), *KCNQ2* (3), *SCN2A* (3), *STXBP1* (2), *NR2F1* (2), *UBA5* (2), and *ABCD1* (2). There were 40 cases (58%) of *de novo* variants in the genetic etiology, 22 cases (31.9%) of genetic variants, and 7 cases (10.1%) without parental verification. There were 7 abnormalities in chromosome tests, including 1 karyotype abnormality in a child with Down syndrome. Identify a specific instance of mitochondrial mutation among the aforementioned genetic variations. The chrM:9,134 spontaneous pathogenic mutation, as demonstrated by Tomas Honzik et al., is associated with clinical symptoms including intrauterine fetal growth retardation, hypotonia, and hypertrophic cardiomyopathy. No definitive correlation exists regarding the development of IESS (15). Therefore, although a mitochondrial variant was present in this patient, the clinical presentation of this variant was not consistent with the clinical presentation of this child (IESS). The most common gene among the single gene variants was *SCN8A* (13.3%), with all cases occurring in females. Furthermore, by functional classification (according to OMIM search classification), genes can be divided into (1) channel classes: electric ion channel related: ① sodium channel related, such as *SCN8A*, *SCN2A*; ② potassium channel related, such as *KCNQ2*, *KCNQ5*, *KCNK4*; ③ calcium channel related, *CACAN2D3*, *CACNA1C*, and ④ ligand-gated channel *GABRA1*; (2) neuronal synapse/receptor classes: *STXBP1*, *PRRT2*, *NR2F1*, and *SPTAN1*; (3) enzyme synthesis-related: *NARS1*, *MECP2*, *UBA5*, *CLU4B*, *WWOX*, *RAB3GAP1*, and *IARS2*; and (4) signaling-related: *GRIN3*, *RAC3*, and *DEPDC5*.
(3) Genetic-structural etiology: genetic-structural etiology were 32 cases (8.9%). In congenital structural causes, 24 cases have pathogenic genes, of which *TSC2* and *NF1* gene variants were the most common, and *PHIP*, *NIN*, *RAB3GAP1*, and other variants were also involved. In acquired structural etiology, 6 cases have pathogenic genes, mainly HIE with *KCQN2*, *RELN*, *SHANK3*, *CACNA2D3*, *RAC3*, and *CHD3* variants.
(4) Metabolic etiology: In the results of plasma amino acids and urine organic acids, only 1 case was abnormal in the 215 cases (60%), which result was fumaric. Based on the imaging results, we diagnosed it as Leigh syndrome. Of the 9 cases of metabolic etiology, 5 cases included glycosylation, 1 case exhibited aberrant sodium metabolism, and 3 cases were connected with mitochondrial mitochondrial encephalopathy-associated epilepsy (Leigh syndrome). Of the 9 cases, 8 cases had received genetic testing, and 6 cases had abnormal results, all exhibiting pathogenic mutations. Among them, *SLC35A2*, *ALG13*, and *ALG2* gene mutations were detected in 5 cases of congenital glycosylation, and in the case of abnormal sodium metabolism, the *AVPR2* gene variant was detected.
(5) Infectious etiology: the infectious etiology was due to intrauterine infection with cytomegalovirus (CMV) in all three cases.
(6) Unknown etiology: Of the 107 cases (64.8%) refined for genetic testing, certain genes were potentially associated, although their pathogenicity remains undetermined. Potentially associated genes: *PDGFRB*, *VPS13D*, *NEXMIF*, *SPTAN1*, *CUX2*, *AP3B2*, *EBF3*, *CACAN1I*, among others.

2. Relationship between etiology and clinical features in each group:
  (1) Figure 1. illustrates the age of onset for several etiologies. The age of onset was 6 (4,8) months for structural etiology, 4 (3,6) months for genetic etiology, 5 (3,7) months for genetic-structural etiology, 5 (2.5,8.5) months for metabolic etiology, a month for infectious etiology, and 5 (4,6.5) months for unknown etiology. Ninety point nine percent (328/361) of patients received intervention within three months after initiation. Family history was observed in 8% (29/361) of patients, with 75.9% (22/29) being immediate family members. There was no statistically significant difference in age of onset, interval between interventions, family history, and type of EEG at early onset between etiologic groups (*p* > 0.05). A statistical disparity existed between the etiological groupings regarding gender, with males exhibiting a greater proportion of structural etiology (*p* = 0.003) and a lesser proportion of genetic-structural (*p* = 0.033) and metabolic etiology (*p* = 0.003) in comparison to females. Regarding full-term or not, structural etiology was more prevalent in preterm infants (*p* < 0.01). The proportion of low birth weight children was higher in structural etiology compared to other etiologies (*p* < 0.001). Regarding the development before onset, a higher percentage of children with structural etiology were developmental regression compared to other etiologies (*p* < 0.001).

(2) The treatment and prognostic classification of the patients are shown in Table 3. The treatments were categorized into ACTH treatment (*n* = 242) and non-ACTH treatment (*n* = 119). Reasons for not receiving ACTH therapy include ① Significant reduction in seizures after application of a single asm; ② Familial collaboration in declining ACTH therapy; ③ Premature cessation of ACTH treatment due to serious side effects (including infection, severe cardiac arrhythmia, hypokalaemia, etc.). The majority of family members of patients who declined ACTH treatment cited the patient's youth, the elevated risks associated with ACTH therapy, and the perception that the apprehension regarding treatment-related suffering outweighed the severity of the disease. More than 60% of patients received treatment with multiple antiseizure medications (ASMs), with topiramate being the most frequently administered (*n* = 341). Vigabatrin (VGB) was utilized in 66 instances, while other ASMs included Valproic acid, levetiracetam, nitrazepam, clonazepam, clobazam, and perampanel. Other treatments included ketogenic diet (*n* = 53), vagus nerve stimulation (VNS) (*n* = 2), and partial lesion removal (*n* = 4). Patients treated with ACTH exhibited a markedly greater proportion of full remission and seizure control relative to those receiving treatment without ACTH (*p* < 0.001). The proportion of hypsarrhythmia resolution following ACTH therapy was greater in patients with an age of onset of 3–6 months (*p* = 0.013), within this group, 5.6% were treated with ACTH only, 94.4% were treated with ACTH and Asms, No significant difference was observed among the three groups of patients aged 3–6 months treated with ACTH alone, and ACTH plus ASMs. Also, we found a higher percentage of the disappearance of hypsarrhythmia in patients with unknown etiology compared to those with genetic etiology in patients with an age of onset of 3–6 months, who were treated by ACTH (*p* = 0.02). However, (*p* = 0.02). However, there was no significant difference in the disappearance of hypsarrhythmia after ACTH treatment among etiology groups at any age of onset. Regarding outcome, there were no ineffective patients among the included cases. A higher percentage of patients with poor outcomes was observed in the structural etiology group (*p* = 0.03) and genetic etiology group (*p* = 0.01) in comparison to the group with unknown etiology. Patients with significant developmental regression before the onset had a worse prognosis (*p* = 0.02). Patients with the disappearance of hypsarrhythmia after ACTH treatment demonstrated a more favorable prognosis (*p* < 0.001). The rest were not statistically different.

**Table 3:** Treatment and prognosis.

Variable	Total patients	Percentage
Treatment		
ACTH	242	67.0%
Non-ACTH	119	33.0%
The hypsarrhythmia disappeared or not after ACTH		
Disappear	181	74.8%
Not disappear	61	25.2%
Prognosis: seizure control		
Complete remission	104	28.8%
Effective	191	52.9%
Poor effect	66	18.3%

ACTH, adrenocorticotropic hormone.

## 5 Discussion

Of the 361 patients with IESS, a definitive etiology was established in 54.2%, predominantly structural, followed by genetic, with metabolic and infectious etiologies representing a minor percentage, and no immunological etiology was discovered. Previous etiological studies of patients with IESS indicate that approximately 60% had a definitive etiological cause, whereas 40% had an unidentified underlying etiological cause. Our research aligns with the conclusions of John P. Osborne, Pan Peng, et al. (8, 9). The predominance of structural etiology indicates the significant function of brain MRI in diagnosing etiology. Lin Wan showed a higher diagnosis rate of 77.1% in her study on the etiology of patients with IESS, which was analyzed and found to be related to the completion of genetic testing (WES 72.3%) (16). This further emphasizes the essential importance of genetic testing in improving diagnosis rates in the future. Numerous researches have identified the prevalent genes associated with IESS as *TSC1*, *TSC2*, *CDKL5*, *ARX*, *KCNQ2*, *STXBP1*, *SCN2A*, *SCN8A*, *NF1*, among others, while common chromosomal abnormalities include Down syndrome (9, 17–19). The primary genes identified in the genetic etiologies of the samples in this investigation were *SCN8A* and *KCNQ2,* which differed from the results reported in previous studies. It may be indicated that the distribution of the genetic etiology may be affected by different regions (6, 8). Among the chromosomal variations, there was only one patient with Down syndrome, markedly differing from the findings of international studies, potentially attributable to the routine implementation of Down syndrome screening and noninvasive DNA testing during pregnancy in China (20).

The research conducted by Karen L. Oliver et al. counted epilepsy-related genes. All gene variants, except for *PHIP*, *NIN*, and *CACNA2D3*, including *SCN8A*, *KCNQ2*, *SCN2A*, and *STXBP1*, were identified within the IESS list of pathogenic genes (21). In this study, there was one case of Dandy-Walker malformation with a pathogenic variation in the *NIN* gene. The *NIN* gene is essential for the maintenance of asymmetric neurogenic divisions of radial glial cells (RGCs) and is linked to neuronal production and number (22). Mutations in the *NIN* gene are associated with malformations of brain structure, which may explain the development of IESS. The *PHIP* gene encodes two protein isoforms: PHIP/DCAF14, which are crucial for neurodevelopment, and *PHIP* is currently identified as a candidate gene in the Intellectual Disability (ID) cohort (23). In recent years, studies have described Chung-Jansen syndrome due to variants in the *PHIP* gene, which is clinically characterized by developmental delay, learning disabilities, behavioral abnormalities, and seizures (24). Our study identified one case of MCD with a pathogenic variant in the *PHIP* gene (exon-40 heterozygous deletion). The patient exhibited developmental regression alongside IESS, while both her mother and maternal grandmother had intellectual disabilities. The validation results showed that the variant was inherited from the mother, potentially elucidating the patient's intellectual disability; however, this did not substantiate a correlation between *PHIP* and IESS. The *PHIP* gene may become a candidate gene for epilepsy in the future. In addition, the link between genotypes and clinical phenotypes of IESS patients was comprehensively analyzed in the study by Pavone et al., and it was mentioned that the phenotypes are closely related to pathogenesis. This further emphasizes the importance of genetic testing and provides new research directions for future individualized treatment for the cause of the disease (25).

**Figure 2:**
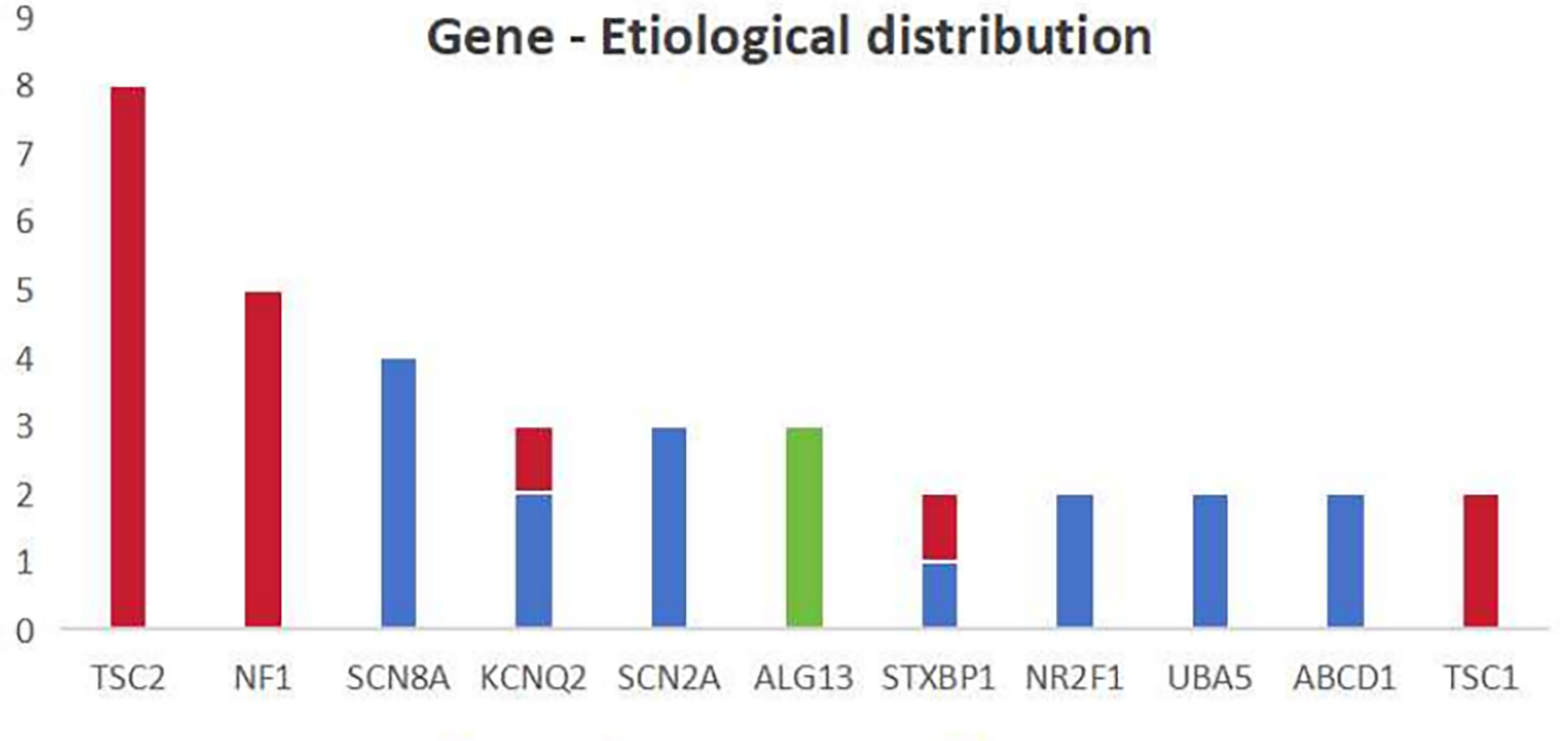
The distribution of different genes among different etiologies the *X*-axis in the chart represents the main types of mutations mentioned in this study and the *Y*-axis represents the number. The blue color represents genetic causes, the red color represents genetic-structural causes and the green color represents metabolic causes.

The distribution of different genes among different etiologies is shown in Figure 2. In our study, four of the 15 patients with tuberous sclerosis complex (TSC) had negative genetic results and one patient had negative brain MRI results. It suggests that the lesions in TSC patients may not be seen on imaging, or that changes in protein function alone can result in the onset of IESS. When genetic and structural etiologies coexist, structural changes are frequently linked to genetic mutations, thus, genetic screening is advised in the presence of typical structural brain changes. Additionally, patients with a metabolic etiology may have negative metabolic screening results. This further emphasizes the importance of genetic testing.

Our study concludes that a higher proportion of males than females are present among patients with IESS with a greater percentage of structural etiology observed in males, preterm infants, low birth weight infants, and patients exhibiting developmental regression prior to onset, consistent with the findings of most contemporary studies (9–11). Simultaneously, certain studies have demonstrated notable disparities between males and females in terms of brain structure, hormonal responses, and response to brain damage treatment, which may explain the above characteristics (26). Studies have shown that patients with significant developmental regression before the onset of IESS have a poorer prognosis, which is consistent with our findings (27).

Regarding the treatment of IESS, ACTH is used as the first-line treatment. The study by Kivity et al. suggests that intervention within one month of onset improves developmental outcomes (28). A comprehensive meta-analysis found that intervention durations of fewer than 4 weeks correlated with improved neurodevelopmental outcomes (29). The study by O'Callaghan et al. also suggests that the epileptic control rate for intervals shorter than 2 months between ACTH interventions was 63%, whereas it was 43% for intervals exceeding 2 months, suggesting that prompt therapy enhances seizure control, which means that early treatment facilitates seizure control (30). An article summarised various relevant studies, and it found the unifying view that early and timely application of first-line drugs for the treatment of IESS is a key factor in improving the prognosis (31). A growing body of research has demonstrated the significance of early hormone therapy for initial seizure control and improvement of prognosis, aligning with our findings. VGB has become the preferred treatment for patients with IESS combined with TSC; however, for non-TSC patients, VGB monotherapy and VGB combined with hormonal therapy do not offer significant benefits over hormonal therapy alone, and VGB is associated with adverse effects, including visual field defects (30, 32–34). This may explain the small proportion of patients who applied VGB therapy in this study. Our investigation revealed that children with peak age onset of the condition exhibited a favorable response to ACTH treatment as evidenced by EEG results, and the duration of the intervention did not correlate with the disappearance of hypsarrhythmia post-ACTH treatment, contradicting the findings of Kelley et al. (35). O'Callaghan FJ et al. suggested that the disappearance of hypsarrhythmia after ACTH treatment may correlate with short-term seizure control and a reduction in recurrence rates. It indicated that patients with epilepsy controlled after ACTH treatment were more likely to have good mental development than those without, suggesting that early epilepsy control and reduction of abnormal discharges may improve developmental outcomes (30, 36, 37). This may explain the favorable prognosis of the patients in this study who had disappearance of hypsarrhythmia after ACTH treatment. An expert consensus suggests that factors of a favorable outcome encompass IESS of unknown etiology, age of onset less than 4 months, and an early and rapid response to treatment, which is consistent with the findings mentioned above (38, 39).

When a patient is diagnosed with IESS, it is essential to ascertain the etiology of IESS promptly to inform the formulation of a tailored treatment plan.

## 6 Conclusion

More than half of the patients with IESS can be identified with pathogenic factors, with structural etiology being the most prevalent, followed by genetic etiology, which includes common pathogenic genes such as TSC2, NF1, SCN8A, and KCNQ2. Gene categories have geographic variability. Males, preterm infants, low birth weight infants, and patients with developmental regression before onset may have a higher likelihood of a structural etiology. Genetic and structural etiology often suggests a poor prognosis, however, patients with unknown etiology may have a good prognosis. Timely diagnosis and concomitant hormone therapy have a positive effect on early seizure control, improved prognosis, and later neurodevelopmental enhancement. Genetic testing is recommended in patients with negative metabolic screening and imaging to identify the etiology of the disease. In addition, the early improvement of genetic testing can help to identify the types of genetic variants as early as possible, and by analyzing the link between genes and clinical phenotypes, it is expected to carry out targeted, individualized diagnosis and treatment in the future.

## 7 Limitations

As this is a retrospective study, the results are prone to bias. This study lacked long-term follow-up data for patients with IESS. In addition, the study population was geographically limited, and the results are only representative of the region studied.

## Data Availability

The raw data supporting the conclusions of this article will be made available by the authors, without undue reservation.
